# Integrating Radiomics with Genomics for Non-Small Cell Lung Cancer Survival Analysis

**DOI:** 10.1155/2022/5131170

**Published:** 2022-08-27

**Authors:** Wei Chen, Xu Qiao, Shang Yin, Xianru Zhang, Xin Xu

**Affiliations:** ^1^School and Hospital of Stomatology, Cheeloo College of Medicine, Shandong University, Shandong Key Laboratory of Oral Tissue Regeneration, Shandong Engineering Laboratory for Dental Materials and Oral Tissue Regeneration, Jinan, China; ^2^Department of Biomedical Engineering, Shandong University, Jinan, China; ^3^School of Mathematics, Shandong University, Jinan, China

## Abstract

**Purpose:**

The objectives of our study were to assess the association of radiological imaging and gene expression with patient outcomes in non-small cell lung cancer (NSCLC) and construct a nomogram by combining selected radiomic, genomic, and clinical risk factors to improve the performance of the risk model.

**Methods:**

A total of 116 cases of NSCLC with CT images, gene expression, and clinical factors were studied, wherein 87 patients were used as the training cohort, and 29 patients were used as an independent testing cohort. Handcrafted radiomic features and deep-learning genomic features were extracted and selected from CT images and gene expression analysis, respectively. Two risk scores were calculated through Cox regression models for each patient based on radiomic features and genomic features to predict overall survival (OS). Finally, a fusion survival model was constructed by incorporating these two risk scores and clinical factors.

**Results:**

The fusion model that combined CT images, gene expression data, and clinical factors effectively stratified patients into low- and high-risk groups. The C-indexes for OS prediction were 0.85 and 0.736 in the training and testing cohorts, respectively, which was better than that based on unimodal data.

**Conclusions:**

Combining radiomics and genomics can effectively improve OS prediction for NSCLC patients.

## 1. Introduction

Non-small cell lung cancer (NSCLC) is one of the deadliest diseases in humans. NSCLC occurs in approximately 80%–85% of lung cancer patients. Surgery is still the only potential cure for patients with early-stage NSCLC. However, 30% to 55% of NSCLC patients will relapse and die of the disease [1]. Therefore, effective prognostic tools are needed to help predict and improve overall survival in patients with NSCLC and to provide specific treatments to improve their quality of life.

Radiomics refers to the extraction of many quantitative image features from radiological data and data mining of these features for clinical tasks, such as disease diagnosis and prognostic analysis [2]. Image features used for extraction and analysis in radiomics include intensity patterns, shape, and a range of texture features [3]. The image analysis is completely computerized, and these features are extracted automatically and with high-throughput [4]. Radiomics in lung cancer has aroused great interest, including application in diagnosis [5] and prognosis analysis [6]. Yang et al. developed a radiomics nomogram by combining the optimized radiomics signatures of CT images and clinical predictors to assess the overall survival of patients with NSCLC [7]. Wang et al. demonstrated that a radiomics signature with multiregional features could help to stratify the survival risk of patients with clinical stage and pathologic stage IA pure-solid NSCLC [8].

With the advancement of high-throughput sequencing technology, using microarray gene expression profiling to analyze gene expression characteristics and establish prognostic gene signatures has also attracted significant research interest. Li et al. established a gene signature closely related to the tumor immune microenvironment that can effectively predict clinical outcomes [9].

The above studies have greatly promoted the discovery of biomarkers for the prognosis of NSCLC. However, most methods are limited to a single-data model, and cross-modal comprehensive methods are relatively underdeveloped. By integrating multimodal data, such as gene expression, radiological and histological imaging, and clinical data, more detailed insights into the development and occurrence of disease can be provided [10]. Therefore, by fusing information from radiological imaging, gene expression, and clinical data, it is possible to significantly improve survival prediction. Recently, this has been demonstrated for recurrence prediction in NSCLC by combining radiological imaging and gene expression data [11].

Multimodal approaches can extract more meaningful features from multiple perspectives, leading to more reliable characterization of tumors, and thus have great potential to address the limitations of single-modality models. According to this assumption, in this study, we tried to provide a complete view of NSCLC characteristics for survival prediction by integrating information from radiological imaging, gene expression, and clinical data. To this end, we first used CT images and gene expression data to develop their respective risk scores and combined them with clinical characteristics to construct the final survival analysis model. We then investigated whether the fusion model could improve the overall survival of NSCLC patients compared with the models based on unimodal data [12].

## 2. Materials and Methods

**Figure 1:**
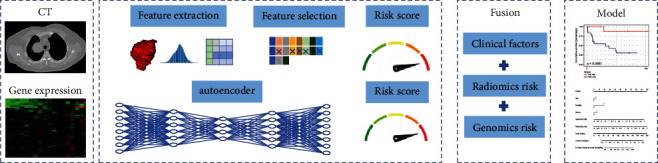
The flowchart of our study.

An overview of our method is depicted in Figure 1. With paired CT and gene expression data, our objective is to develop multimodal representation from both modalities that would outperform unimodal representations in NSCLC survival prediction. For CT images, we followed the conventional radiomics research pipeline, which mainly includes feature extraction, feature selection, and model analysis. For gene expression data, we adopted a deep learning architecture to extract the latent features that best represent the data. For CT and gene expression data, we calculated their respective risk scores. Finally, we fused these risk scores with clinical data to obtain the final prognostic analysis model.

### 2.1. Problem Formulation

Let (*x*_*i*_, *t*_*i*_, *δ*_*i*_) denote each patient, where *x*_i_ corresponds to the features extracted from CT or gene expression, *t*_*i*_ is the survival time, and *δ*_*i*_ denotes the censoring indicator. *δ*_*i*_=1 means a noncensored instance and vice versa. The purpose of survival analysis is to predict the time duration until a specific event occurs. In this study, the event was the death of a patient with NSCLC. The Cox proportional hazard model is one of the most popular methods to model the hazards function [13]. It is built based on the hypothesis that the hazard ratio between two instances is time-independent and is defined as

(1) $$\begin{array}{c} h\left(t|x\right)=h_{0}\left(t\right)\exp\left(\alpha^{T}x\right), \end{array}$$

where *h*_0_(*t*) corresponds to the baseline hazard and *f*(*x*)=*α*^*T*^*x* is the risk function. *α*=(*α*^1^, *α*^2^,…*α*^*υ*^) is the regression parameter that can be estimated by minimizing the following log partial likelihood function:

(2) $$\begin{array}{c} l\left(\alpha \right)=-\sum_{i=1}^{N}\delta_{i}\left(\alpha^{T}x_{i}-\log\sum_{j\in R\left(t_{i}\right)}\exp\left(\alpha^{T}x_{j}\right)\right), \end{array}$$

where *N* denotes the number of patients and *R*(*t*_*i*_) is the risk set at time *t*_*i*_, which represents the set of patients who are still at risk before time *t*_*i*_.

### 2.2. Data Source

We used the NSCLC Radiogenomics dataset, a public dataset of 211 patients who underwent surgery for NSCLC between 2008 and 2012 [14, 15]. It includes two cohorts from Stanford University School of Medicine (AMC) and Palo Alto Veterans Affairs Healthcare System (R01). CT scans were performed using different scanners and imaging protocols, with slice thicknesses ranging from 0.625 to 3.0 mm (median, 1.5 mm), an X-ray tube current of 124–699 mA (mean, 220 mA), tension of 80–140 kV (mean, 120 kV), and a field of view after manual shortening ranging from 71 to 124 cm (mean 89 cm) [16]. The corresponding gene expression data were downloaded from Gene Expression Omnibus datasets (GEO; GSE103584), which are composed of RNA sequences (RNA-seq) described by fragments per kilobase of transcript per million mapped reads (FPKM) of 130 NSCLC patients. We selected samples with paired CT scans and gene expression data, and a total of 116 samples were included in the study.

### 2.3. Construction of the Radiomics Model

For each CT image, we extracted a wide range of features from the segmented cancer region according to the radiomic features described by the Imaging Biomarker Standardization Initiative (IBSI), including intensity features, shape features, texture features, and wavelet features [8]. Intensity features use first-order statistics, such as energy and entropy, to quantify the tumor intensity characteristics. Shape features describe the shape characteristics of the tumor, such as sphericity or compactness. Texture features can quantify intratumor heterogeneity by taking the spatial location of each voxel compared with the surrounding voxels into account. Commonly used texture features include gray level cooccurrence matrix (GLCM), gray level run length matrix (GLRLM), gray level size zone matrix (GLSZM), neighbouring gray tone difference matrix (NGTDM), and gray level dependence matrix (GLDM). Wavelet features calculate the intensity and textural features from wavelet decompositions to represent the tumor characteristics at different frequencies. All feature extraction algorithms were implemented in the Pyradiomics toolkit [17]. A detailed description of these features can be found in the Supplementary Appendix.

To eliminate the differences in the value scales of the radiomic features, feature normalization was performed after feature extraction. For the extracted features in the training cohort, each feature for a specific patient was subtracted by the mean value and divided by the standard deviation value from this cohort. The same normalization method was applied to features in the testing cohort using the mean and standard deviation values calculated based on the training cohort.

Including too many features will increase the computational cost, and redundancy between features will reduce the accuracy of the model. Furthermore, the number of features is more than the number of samples in this work, which will increase the probability of overfitting. Therefore, we needed to select a small number of informative radiomics features to predict the survival risk of patients. In this study, we used the least absolute shrinkage and selection operator (Lasso) [18] for feature selection.

The Lasso can shrink some regression coefficients to zero and select important variables by adding an L_*i*_ regularization term to *l*(*α*), so the Lasso-Cox model can be formulated as

(3) $$\begin{array}{c} \min\left\{l\left(\alpha \right)+\lambda \sum_{k=1}^{p}\left|\alpha_{k}\right|\right\}, \end{array}$$

where ∑_*k*=1_^*p*^|*α*_*k*_| is the L_1_ regularization term and *λ* is a parameter to balance the two parts. We used 5-fold cross-validation to determine the optimal value of *λ*.

### 2.4. Construction of Genomics Model

We chose the autoencoder framework for processing gene expression data. Autoencoders aim to reconstruct the input values using combinations of nonlinear functions, and the bottleneck layers are considered as the representation of the inputs [19]. Usually, the bottleneck layer has significantly fewer neural units than the input layer, and thus can be regarded as a compressed representation of the original input. Autoencoders have already been shown to be efficient for dimension reduction of high-dimensional genomics data [20].

An autoencoder can be divided into an encoder and a decoder. Let the original input be *X* ∈ *R*^*N*×*p*^, with *N* samples and *p* features. We used a multilayer neural network with parameter Φ_*e*_ as the encoder:

(4) $$\begin{array}{c} \text{Encoder}\left(X,\phi_{e}\right)=H\in R^{N\times k}. \end{array}$$

*H* is regarded as a compressed representation of input *X*. The encoder maps *N* samples from *p*-dimension space to *k*-dimension space. Another multilayer *r* neural network with parameter Φ_*d*_ is regarded as the decoder:

(5) $$\begin{array}{c} \text{Decoder}\left(X,\phi_{d}\right)=\tilde{X}\in R^{N\times p}, \end{array}$$

where is $\tilde{X}$ reconstruction representation which has the same shape as *X*. The decoder maps *N* samples from *k*-dimensional space back to *p*-dimensional space. The whole process of the autoencoder can be expressed as

(6) $$\begin{array}{c} \text{Decoder}\left(\text{Encoder}\left(X,\phi_{e}\right),\phi_{d}\right)=\tilde{X}. \end{array}$$

The parameters of the autoencoder can be estimated by minimizing the following reconstruction error:

(7) $$\begin{array}{c} \underset{\phi_{e},\phi_{d}}{\arg\min}{\left\|X-\tilde{X}\right\|}_{F}^{2}. \end{array}$$

Then, the latent representation *H* can be regarded as the compressed form of *X* that can be used in subsequent tasks.

### 2.5. Validation of Radiomics and Genomics Models

For the features selected from CT and gene expression data, we constructed Cox models. Therefore, a risk score of each patient can be computed by ∑_*i*=1_^*k*^*β*_*i*_ × *f*_*i*_, where *f*_*i*_ denotes the selected radiomic features or the compressed gene features, and *β*_*i*_ represents the estimated coefficient of the corresponding features. According to this formula, the risk score of each patient was computed. Then, all patients in the training cohort were divided into high- and low-risk groups with the median of the risk score as the cutoff. Then, the survival differences between these two groups were evaluated by a Kaplan–Meier (KM) survival curve. We used the same category for the testing cohort. Accordingly, the samples in the testing cohort were also divided into two risk groups. The difference in the survival curves of the high-risk and low-risk groups was evaluated by the log-rank test.

### 2.6. Construction of a Nomogram Combining Two Risk Scores and Clinical Factors

The candidate prognostic indicators included age, sex, stage, grade, and the above two risk scores. We first used a LASSO Cox regression model to select the final features for constructing the fusion model. Then, a nomogram was built using a multivariate Cox proportional hazard model. We used the same validation methods as described in the validation of the radiomics and genomics models.

### 2.7. Statistical Analysis

The statistical analysis was performed with R software, version 4.1.2, and Python software, version 3.8.5. Cox regression was performed using the “scikit-survival” package [21]. Nomograms were generated using the “rms” package. The differences in clinical factors between the training and testing cohorts were assessed using the “tableone” package [22].

## 3. Results and Discussion

### 3.1. Clinical Characteristics

We randomly split the dataset, including 87 training and 29 testing samples. The clinical characteristics in the training and testing cohorts are summarized in Table 1. There was no significant difference in age, gender, N stage, *M* stage, or grade between the training and independent testing cohorts (*P* > 0.05).

### 3.2. Construction and Validation of Radiomics Model

**Figure 2:**
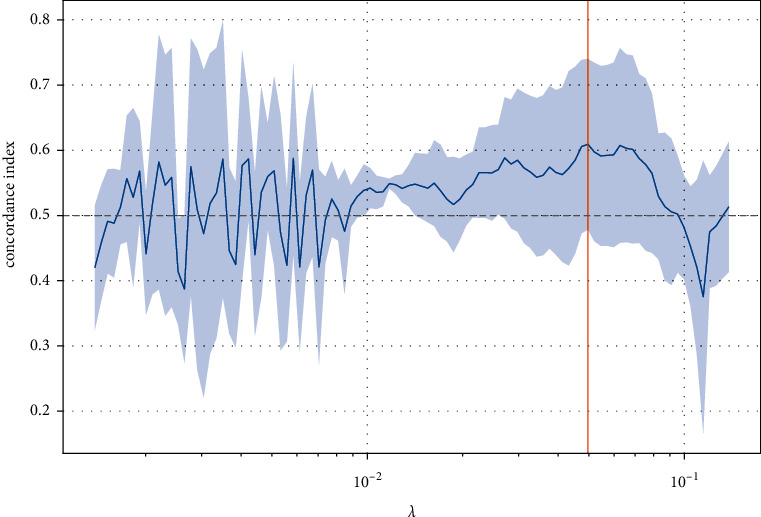
Feature selection using the LASSO Cox regression model.

There were 8 features with nonzero coefficients in the LASSO Cox regression model. The optimal *λ* selection in the LASSO Cox regression model is shown in Figure 2. The radiomic signature based on the eight features and the weight for each feature are given in Table 2.

**Figure 3:**
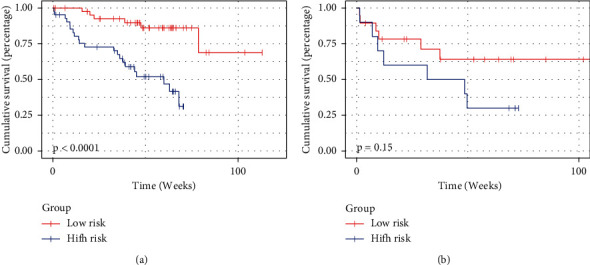
Survival prediction of the risk score calculated from CT-based radiomics signatures. (a) Kaplan–Meier curves of the training cohort. (b) Kaplan–Meier curves of the testing cohort.

The radiomics signature achieved a C-index of 0.79 for the training cohort, and 0.643 for the testing cohort, demonstrating the predictive performance of the model. Based on the risk score of patients in the training cohort, the optimal cutoff was −0.150. Then, patients in both the training and testing cohorts were stratified into low-risk and high-risk groups, as shown in Figure 3. The association of the radiomics signature with OS was shown in the training cohort (*P* < 0.001) and confirmed in the testing cohort (*P*=0.045).

### 3.3. Construction and Validation of Genomics Model

**Figure 4:**
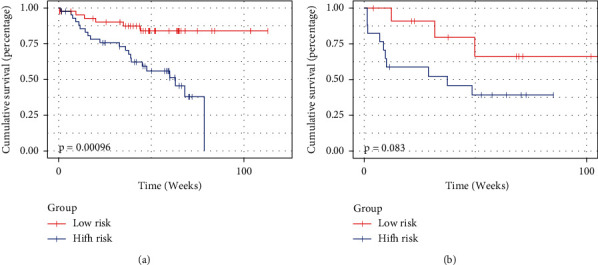
Survival prediction of risk score calculated from genomics signatures. (a) Kaplan–Meier curves of the training cohort. (b) Kaplan–Meier curves of the testing cohort.

In the autoencoder structure, the number of latent features is an important parameter. To determine this parameter, we preset the number of latent features to 5–12. For each set of potential features, we further compressed it into two dimensions using multidimensional scaling (MSD) [23], as well as the original gene, and then calculated the Euclidean distance between the two projections. The experimental results showed that 10 dimensions had the lowest Euclidean distance, so we set the number of hidden features to 10. The C-index of the genomics risk score was 0.716 for the training cohort and 0.581 for the testing cohort. Based on the risk score of patients in the training cohort, the optimal cutoff was −0.006. Then, patients in both the training and testing cohorts were stratified into low-risk and high-risk groups, as shown in Figure 4. The association of the radiomics signature with OS was shown in the training cohort (*P* < 0.001) and confirmed in the testing cohort (*P*=0.083).

### 3.4. Nomogram that Combines Multimodality

We used LASSO analysis to select the optimal feature combination in the multimodal analysis. A combination of the radiomics risk score, genomics risk score, age, *N* grade, and stage was finally selected.

**Figure 5:**
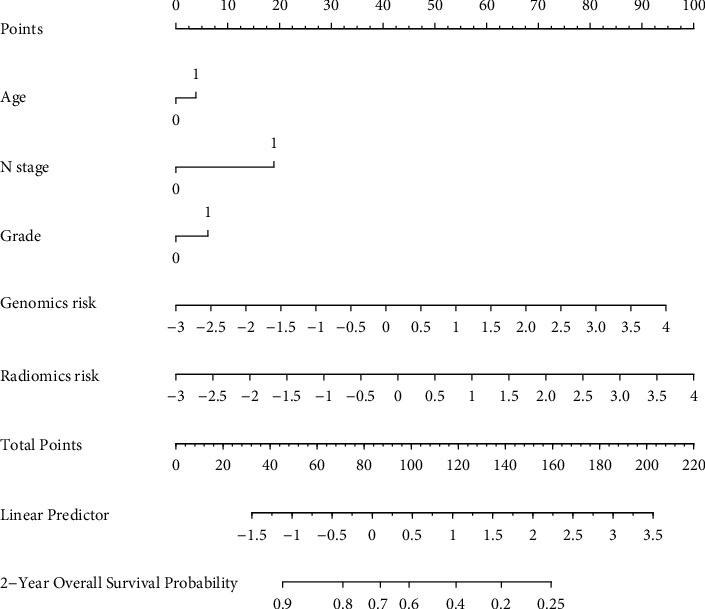
Nomogram for the prediction of 2-year survival.

**Figure 6:**
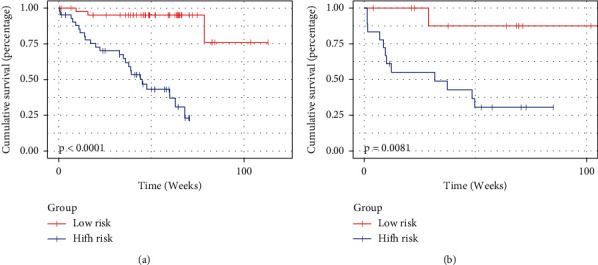
Survival prediction of risk score calculated from the fusion model. (a) Kaplan–Meier curves of the training cohort. (b) Kaplan–Meier curves of the testing cohort.

The combination nomogram for the prediction of 2-year survival is displayed in Figure 5. The multimodal-based model obtained a C-index of 0.85 in the training cohort and 0.736 in the testing cohort. It is worth noting that the C-index of the fusion model was the highest compared to that of the other models in all cohorts. Furthermore, the fusion model obtained the best prognostic ability in stratifying patients into high-risk and low-risk groups with *p*=0.0081, as shown in Figure 6. In Table 3, we list the results of all models.

To further verify the effectiveness of our model, we performed 5-fold cross-validation for all data. All patients were randomly divided into 5 subsamples of equal size, and in each fold, 4 subsamples were used as training data and the remaining 1 subsample was used as validation data for testing. For each fold, we used the method presented in this paper separately. The average and standard deviation of the C-indexes over the 5-fold cross-validation are listed in Table 3. As can be seen from the results, the C-indexes of the 5-fold cross-validation were consistent with the results of the testing cohort, proving the stability of our model.

## 4. Discussion

Gene expression data, imaging data and clinical factors each play an important role in the diagnosis and prognosis of diseases. Merging these multimodal data may lead to new prognostic cancer models and provide new support for patient treatment strategies. Accordingly, increased attention is given to statistical methods and algorithms to analyze and correlate multivariate imaging, clinical and gene expression data for disease diagnosis and prognosis. Our research demonstrated the ability to integrate radiomics data, genomics data, and clinical features for the stratification of lung cancer patients. Unimodal analysis shows that only using gene expression data, radiology data, or clinical features cannot effectively stratify patients. With unimodal data, the difference in survival between the two risk groups in the testing group was not significant (*p*=0.15 for radiomics model, *p*=0.08 for genomics model, and *p*=0.015 for clinical features model). When using age, N stage, grade, radiomics risk score, and genomics risk score, the model can significantly distinguish high-risk and low-risk groups (*p*=0.0081).

This study has some limitations. First, all the samples we explored are public and the sample size is relatively small, limiting the use of more advanced methods. Second, for molecular data, we only used gene expression data. More meaningful discoveries may be produced if data such as miRNA and DNA methylation data are fused. Third, we only discussed the handcrafted radiomic features. Many studies have shown that features based on deep learning have better predictive capabilities. We believe that the fusion of multiple modalities can help detect more effective biomarkers and improve lung cancer clinical decision-making. In future work, we will solve the above limitations and conduct further multicenter, large-scale researches to promote the application of multimodal fusion in the management of lung cancer patients.

## 5. Conclusions

In this study, the risk score developed based on multimodal data has great potential to improve the determination of overall survival of NSCLC patients compared with the models based on unimodal data. We demonstrated that we could gain more significant insights into cancer prognosis by fusing imaging and genomic data. More studies that combine more data sources, such as multiomics data and digital pathology, are required to confirm the advantages of multimodal fusion.

## Figures and Tables

**Table 1:** Characteristics of patients in the training and testing cohorts.

Characteristic	Training cohort (*N* = 87)	Testing cohort (*N* = 29)	*p*
Age (years)			
>60	59	17	0.3669
<60	28	12	

Gender			
Female	22	7	0.9015
Male	65	22	

N stage			
N0	71	25	0.3854
≥N1	19	4	

M stage			
M0	85	27	0.2399
M1	2	2	

Grade			
0	64	19	0.6251
1	27	10	

**Table 2:** Description and Cox proportional hazard weights of each feature in the radiomic signature.

Feature name	Feature type	Weight
ClusterProminence	GLCM	1.0590
LargeDependenceLowGrayLevelEmphais	GLDM	0.8589
Range	Firstorder	−1.3498
LargeAreaEmphasis	GMSZM	−1.0405
SizeZoneNonUniformity	GMSZM	−0.9399
Idmn	GLCM	−0.5833
MajorAxisLength	Shape	2.0625
LargeDependenceHighGrayLevelEmphasis	GLDM	1.3778

**Table 3:** The results of all models.

	Training cohort		Testing cohort		Cross validation
	C-index	*p* value	C-index	*p* value	C-index
Radiomics	0.79	<0.0001	0.643	0.045	0.653 ± 0.028
Genomics	0.716	0.00096	0.581	0.083	0.606 ± 0.081
Fusion model	0.85	<0.0001	0.736	0.0081	0.749 ± 0.044

## Data Availability

Public data can be freely accessed and downloaded at https://wiki.cancerimagingarchive.net/display/Public/NSCLC+Radiogenomics.

## Supplementary Materials

Supplementary Materials Supplementary Table S1: first-order features. Supplementary Table S2: shape features. Supplementary Table S3: gray level co-occurrence matrix (GLCM) features. Supplementary Table S4: gray level size zone matrix (GLSZM) features. Supplementary Table S5: gray level run length matrix (GLRLM) features. Supplementary Table S6: neighbouring gray tone difference matrix (NGTDM) features. Supplementary Table S7: gray level dependence matrix (GLDM) features.

## References

[1] Uramoto H., Tanaka F. (2014). Recurrence after surgery in patients with NSCLC. *Translational Lung Cancer Research*.

[2] Lambin P., Rios-Velazquez E., Leijenaar R. (2012). Radiomics: extracting more information from medical images using advanced feature analysis. *European Journal of Cancer*.

[3] Han F., Wang H., Zhang G. (2015). Texture feature analysis for computer-aided diagnosis on pulmonary nodules. *Journal of Digital Imaging*.

[4] Kumar V., Gu Y., Basu S. (2012). Radiomics: the process and the challenges. *Magnetic Resonance Imaging*.

[5] Yang F., Chen W., Wei H. (2020). Machine learning for histologic subtype classification of non-small cell lung cancer: a retrospective multicenter radiomics study. *Frontiers in Oncology*.

[6] Wei H., Yang F., Liu Z. (2019). Application of computed tomography‑based radiomics signature analysis in the prediction of the response of small cell lung cancer patients to first‑line chemotherapy. *Experimental and Therapeutic Medicine*.

[7] Yang L., Yang J., Zhou X. (2019). Development of a radiomics nomogram based on the 2D and 3D CT features to predict the survival of non-small cell lung cancer patients. *European Radiology*.

[8] Wang T., She Y., Yang Y. (2021). Radiomics for survival risk stratification of clinical and pathologic stage IA pure-solid non-small cell lung cancer. *Radiology*.

[9] Li J., Wang H., Li Z. (2020). A 5-gene signature is closely related to tumor immune microenvironment and predicts the prognosis of patients with non-small cell lung cancer. *BioMed Research International*.

[10] Boehm K. M., Khosravi P., Vanguri R., Gao J., Shah S. P. (2021). Harnessing multimodal data integration to advance precision oncology. *Nature Reviews Cancer*.

[11] Subramanian V., Do M. N., Syeda-Mahmood T. Multimodal fusion of imaging and genomics for lung cancer recurrence prediction.

[12] Amini M., Nazari M., Shiri I. (2021). Multi-level multi-modality (PET and CT) fusion radiomics: prognostic modeling for non-small cell lung carcinoma. *Physics in Medicine and Biology*.

[13] Lin D. Y., Wei L. J. (1989). The robust inference for the Cox proportional hazards model. *Journal of the American Statistical Association*.

[14] Clark K., Vendt B., Smith K. (2013). The Cancer Imaging Archive (TCIA): maintaining and operating a public information repository. *Journal of Digital Imaging*.

[15] Bakr S., Gevaert O., Echegaray S. (2018). A radiogenomic dataset of non-small cell lung cancer. *Scientific Data*.

[16] Blanc-Durand P., Campedel L., Mule S. (2020). Prognostic value of anthropometric measures extracted from whole-body CT using deep learning in patients with non-small-cell lung cancer. *European Radiology*.

[17] van Griethuysen J. J. M., Fedorov A., Parmar C. (Nov 1 2017). Computational radiomics System to decode the radiographic phenotype. *Cancer Research*.

[18] Wang W., Liu W. (2020). Integration of gene interaction information into a reweighted Lasso-Cox model for accurate survival prediction. *Bioinformatics*.

[19] Chaudhary K., Poirion O. B., Lu L., Garmire L. X. (2018). Deep learning-based multi-omics integration robustly predicts survival in liver cancer. *Clinical Cancer Research*.

[20] Chen L., Cai C., Chen V., Lu X. (2016). Learning a hierarchical representation of the yeast transcriptomic machinery using an autoencoder model. *BMC Bioinformatics*.

[21] Pölsterl S. (2020). Scikit-survival: a library for time-to-event analysis built on top of scikit-learn. *Journal of Machine Learning Research*.

[22] Tan Y., Mu W., Wang X. C., Yang G. Q., Gillies R. J., Zhang H. (2019). Improving survival prediction of high-grade glioma via machine learning techniques based on MRI radiomic, genetic and clinical risk factors. *European Journal of Radiology*.

[23] Cox M. A., Cox T. F. (2008). Multidimensional scaling. *Handbook of Data Visualization*.

