# Supplementary material for: Integrating Radiomics with Genomics for Non-Small Cell Lung Cancer Survival Analysis
Source: J Oncol. 2022 Aug 27;2022:5131170. doi: 10.1155/2022/5131170 (PMC9440821; doi:10.1155/2022/5131170)
Supplement: Supplementary Materials — Supplementary Table S1: first-order features. Supplementary Table S2: shape features. Supplementary Table S3: gray level co-occurrence matrix (GLCM) features. Supplementary Table S4: gray level size zone matrix (GLSZM) features. Supplementary Table S5: gray level run length matrix (GLRLM) features. Supplementary Table S6: neighbouring gray tone difference matrix (NGTDM) features. Supplementary Table S7: gray level dependence matrix (GLDM) features. [file 5131170.f1.docx]

For each CT image, we extracted a wide range of features from the segmented cancer region according to the radiomic features described by the Imaging Biomarker Standardization Initiative (IBSI), including intensity features, shape features, texture features, and wavelet features. These features are listed in Table S1-S7.

Table S1. First-order features

| Feature names and abbreviations |
| --- |
| **Energy** |
| **Total Energy** |
| **Entropy** |
| **Minimum** |
| **10th percentile** |
| **90th percentile** |
| **Maximum** |
| **Mean** |
| **Median** |
| **Interquartile Range** |
| **Range** |
| **Mean Absolute Deviation (MAD)** |
| **Robust Mean Absolute Deviation (rMAD)** |
| **Root Mean Squared (RMS)** |
| **Standard Deviation** |
| **Skewness** |
| **Kurtosis** |
| **Variance** |
| **Uniformity** |

Table S2. Shape features

| Feature names and abbreviations |
| --- |
| Mesh Volume |
| Voxel Volume |
| Surface Area |
| Surface Area to Volume ratio |
| Sphericity |
| Compactness 1 |
| Compactness 2 |
| Spherical Disproportion |
| Maximum 3D diameter |
| Maximum 2D diameter (Slice) |
| Maximum 2D diameter (Column) |
| Maximum 2D diameter (Row) |
| Major Axis Length |
| Minor Axis Length |
| Least Axis Length |
| Elongation |
| Flatness |

Table S3. Gray level co-occurrence matrix (GLCM) features

| Feature names and abbreviations |
| --- |
| Autocorrelation |
| Joint Average |
| Cluster Prominence |
| Cluster Shade |
| Cluster Tendency |
| Contrast |
| Correlation |
| Difference Average |
| Difference Entropy |
| Difference Variance |
| Joint Energy |
| Joint Entropy |
| Informational Measure of Correlation (IMC) 1 |
| Informational Measure of Correlation (IMC) 2 |
| Inverse Difference Moment (IDM) |
| Maximal Correlation Coefficient (MCC) |
| Inverse Difference Moment Normalized (IDMN) |
| Inverse Difference (ID) |
| Inverse Difference Normalized (IDN) |
| Inverse Variance |
| Maximum Probability |
| Sum Average |
| Sum Entropy |
| Sum of Squares |

Table S4. Gray level sizezone matrix (GLSZM) features

| Feature names and abbreviations |
| --- |
| Small Area Emphasis (SAE) |
| Large Area Emphasis (LAE) |
| Gray Level Non-Uniformity (GLN) |
| Gray Level Non-Uniformity Normalized (GLNN) |
| Size-Zone Non-Uniformity (SZN) |
| Size-Zone Non-Uniformity Normalized (SZNN) |
| Zone Percentage (ZP) |
| Gray Level Variance (GLV) |
| Zone Variance (ZV) |
| Zone Entropy (ZE) |
| Low Gray Level Zone Emphasis (LGLZE) |
| High Gray Level Zone Emphasis (HGLZE) |
| Small Area Low Gray Level Emphasis (SALGLE) |
| Small Area High Gray Level Emphasis (SAHGLE) |
| Large Area Low Gray Level Emphasis (LALGLE) |
| Large Area High Gray Level Emphasis (LAHGLE) |

Table S5. Gray level run length matrix (GLRLM) features

| Feature names and abbreviations |
| --- |
| Short Run Emphasis (SRE) |
| Long Run Emphasis (LRE) |
| Gray Level Non-Uniformity (GLN) |
| Gray Level Non-Uniformity Normalized (GLNN) |
| Run Length Non-Uniformity (RLN) |
| Run Length Non-Uniformity Normalized (RLNN) |
| Run Percentage (RP) |
| Gray Level Variance (GLV) |
| Run Variance (RV) |
| Run Entropy (RE) |
| Low Gray Level Run Emphasis (LGLRE) |
| High Gray Level Run Emphasis (HGLRE) |
| Short Run Low Gray Level Emphasis (SRLGLE) |
| Short Run High Gray Level Emphasis (SRHGLE) |
| Long Run Low Gray Level Emphasis (LRLGLE) |
| Long Run High Gray Level Emphasis (LRHGLE) |

Table S6. Neighbouring gray tone difference matrix (NGTDM) features

| Feature names and abbreviations |
| --- |
| Coarseness |
| Contrast |
| Busyness |
| Complexity |
| Strength |

Table S7. Gray level dependence matrix (GLDM) features

| Feature names and abbreviations |
| --- |
| **Small Dependence Emphasis (SDE)** |
| **Large Dependence Emphasis (LDE)** |
| **Gray Level Non-Uniformity (GLN)** |
| **Dependence Non-Uniformity (DN)** |
| **Dependence Non-Uniformity Normalized (DNN)** |
| **Gray Level Variance (GLV)** |
| **Dependence Variance (DV)** |
| **Dependence Entropy (DE)** |
| **Low Gray Level Emphasis (LGLE)** |
| **High Gray Level Emphasis (HGLE)** |
| **Small Dependence Low Gray Level Emphasis (SDLGLE)** |
| **Small Dependence High Gray Level Emphasis (SDHGLE)** |
| **Large Dependence Low Gray Level Emphasis (LDLGLE)** |
| **Large Dependence High Gray Level Emphasis (LDHGLE)** |
